# Cardiovascular System is Influenced by Skeletal Muscle-derived Extracellular Vesicles, Myokines and MicroRNAs Based on Interorgan Communication: A Systematic Review

**DOI:** 10.7150/ijms.111775

**Published:** 2025-04-28

**Authors:** Jiaxin Chen, Jingxin Zong, Sha Su, Xiang Ji, Lei Wang, Xiaowan Han, Mingjing Zhao

**Affiliations:** 1 Key Laboratory of Chinese Internal Medicine of Ministry of Education and Beijing, Dongzhimen Hospital Beijing University of Chinese Medicine, Beijing, China.; 2 Department of Cardiology, Dongzhimen Hospital Beijing University of Chinese Medicine, Beijing, China.

**Keywords:** skeletal muscle, heart, blood vessels, interorgan communication, bioactive molecules, myokines

## Abstract

**Background/Aims:** To illustrate the types and roles of skeletal muscle-derived bioactive molecules in mediating the communication from skeletal muscle to the heart and blood vessels.

**Methods:** A systematic literature search was performed in four different databases. Eligible articles were screened using the inclusion and exclusion criteria. Two researchers independently performed literature screening and selection, data extraction and literature quality analysis.

**Results:** This study included 29 articles (2 clinical studies and 27 basic studies). Data analysis of the included studies revealed that skeletal muscle synthesizes and releases abundant extracellular vesicles (EVs), myokines (FSTL1, FNDC5/irisin and others) and microRNAs (miRNA-126 and others) to mediate the communication from skeletal muscle to the heart and blood vessels. Certain skeletal muscle-derived EVs, myokines and miRNAs were found to enhance cardiac function, reduce cardiac fibrosis and inhibit cardiac injury, and improve apoptosis and inflammation. In the blood vessels, these bioactive molecules stimulated angiogenesis, improved endothelial cell function, protected against vascular stiffness, and attenuated atherosclerosis and neointimal hyperplasia. Notably, IL-10, FSTL1, b-FGF, VEGF, irisin, musclin, myonectin, exo-miRNA26a, and miRNA-126 definitely played protective roles in the heart and blood vessels through interorgan communication.

**Conclusion:** Skeletal muscle synthesizes and releases EVs, myokines and miRNAs, which mediate the communication from skeletal muscle to the heart and blood vessels. The majority of these bioactive molecules are associated with cardiovascular protective effects. And they may provide new targets for more in-depth mechanism and clinical researches of communication from skeletal muscle to the heart and blood vessels.

## Introduction

Globally, cardiovascular diseases (CVDs) are the leading cause of death in humans. Furthermore, the end-stage of CVDs often manifests as multi-organ and multi-systemic disorder, including skeletal muscle 1, 2. The majority of CVDs patients are accompanied by skeletal muscle wasting and exercise intolerance. Similarly, muscle wasting is associated with higher cardiovascular mortality 3, 4, but the mechanism remains unclear. As is well known, exercise training attenuates muscle wasting 5, 6 and promotes cardiac rehabilitation in CVDs patients 7, 8. The beneficial effects of exercise training may be related to the synthesis and release of bioactive molecules during rapid contraction of the muscle fibers. Skeletal muscle is the largest organ in the human body. Previously, it was regarded merely as a locomotor organ. However, after 2000, skeletal muscle was gradually considered as an endocrine organ because *A. Steensberg* discovered that skeletal muscle secreted abundant interleukin (IL)-6 after exercise in human 9. Later, the concept of myokines was proposed.

Myokines are cytokines and other peptides produced, expressed, and released by muscle fibers. They exert autocrine, paracrine and endocrine effects 10. Skeletal muscle secretes abundant extracellular vesicles (EVs), myokines and microRNAs (miRNAs) induced by exercise to exert on the heart and blood vessels 11, 12. This is a novel mechanism by which exercise protects cardiovascular system. Though a lot of researches related to myokines has not focused on EVs, emerging evidence demonstrates that the majority of them are transported via EVs. In other words, EVs are a crucial vehicle in mediating interorgan communication 13, 14. Therefore, skeletal muscle cells can be regarded as donor cells that alter the physiological and pathological conditions in the recipient cells through bioactive molecules with EVs. Although there is no direct physical connection between the heart and the skeletal muscle, skeletal muscle-derived EVs, myokines and miRNAs act on the heart and the blood vessels through interorgan communication and paracrine pathways. Thus, it is essential to summarize the mechanisms and roles by which the skeletal muscle communicates with the heart or blood vessels under pathological and physiological conditions.

The sources of myokines are multifaceted and complex. Most of myokines are primarily secreted by the skeletal muscle, but they are also secreted by other tissues or organs. Previous review 12 articles about myokines did not explicitly describe that the source of myokines was skeletal muscle and that the target organs were the heart or blood vessels. Moreover, it did not involve in EVs and miRNAs. This article investigates the mechanisms and roles of bioactive molecules secreted by skeletal muscle on the heart or blood vessels under various cardiovascular disease models. It for the first time systematically summarizes that skeletal muscle-derived EVs, myokines and miRNAs modulate the functional and structural status of the heart and blood vessels through interorgan communication.

## Methods

This systematic review was based on the PRISMA 2020 Statement.

### Information Sources and Search Strategy

The published articles were searched comprehensively in four electronic databases (PubMed, Embase, Web of Science and Cochrane Library) from inception to June 16, 2024. To collect as much data as possible, we did not restrict the language of the articles. The keywords and Medical Subject Heading (MeSH) terms used in these searches were as follows: ((Cardiovascular Diseases) OR (Cardiovascular System)) AND (Muscle, Skeletal) AND (Extracellular Vesicles, miRNAs, Myokines, musclin/ostn, irisin/FNDC5, IL-6, IL-8, IL-10, IL-15, IL-1β, IL-1α, SPARC, OSM, Fibroblast Growth Factor 21, Fibroblast Growth Factor 2, Decorin, Myostatin, Leukemia Inhibitory Factor, myonectin, Apelin, Follistatin-Related Proteins, Angiopoietin-Like Protein 4, Growth Differentiation Factor 15, Chemokine CX3CL1, Chemokine CXCL10, Chemokine CXCL12, Osteoglycin, Chitinase-3-Like Protein 1, erythroferrone, Ciliary Neurotrophic Factor, Metrn, BRINP3, Ctsb, Ketoglutaric Acids, MG53). The searches were performed without any filtering. The specific study retrieval strategy is described in the Supplement 1. Subsequently, the references of the extracted studies and reviews were further searched to identify more relevant studies.

### Inclusion and Exclusion Criteria

The inclusion criteria for the clinical studies and the basic studies were as follows: (1) Articles regarding EVs, myokines or miRNAs secreted by the skeletal muscle, including using skeletal muscle-specific gene knockout or overexpression, intramuscular injection of recombinant protein or plasmid and changes of expression level of bioactive molecules in skeletal muscle through other stimulation. (2) Articles regarding bioactive molecules targeting the heart or blood vessels; (3) Clinical trials, animal studies, and cell studies; (4) Articles without language restriction; studies published in the official journals.

The exclusion criteria were as follows: (1) Articles with incomplete information; (2) Experimental data with detection of bioactive molecules only in blood and absence of data from the skeletal muscle tissue and cells.

### Study Selection and Data Extraction

Titles and abstracts of all the articles were assessed independently by two researchers (JX.C. and JX.Z.) based on the inclusion and exclusion criteria. Firstly, the initial results were analyzed and duplicate articles from different databases were removed. Secondly, according to inclusion and exclusion criteria, irrelevant articles were eliminated after reading the title and the abstract. Finally, the full texts of the remaining articles were evaluated and the relevant studies were included.

Two authors (JX.C. and JX.Z.) individually extracted data from the included literature using a standardized sheet prepared for this review. The extracted data included author, year, journal, country, sources of bioactive molecules, ways of producing bioactive molecules, objects of study, models/diseases, targets of the bioactive molecules, whether carried by EVs or not, and the effect of bioactive molecules on the heart and blood vessels. Any disagreement between the two authors was resolved by discussion and consultation with the corresponding authors (MJ.Z. and XW.H.).

### Assessment of Bias Risk and Study Quality

Quality assessment of the included animal studies was systematically performed using SYRCLE's Risk of Bias Tool 15 a validated instrument specifically designed for preclinical animal research. This comprehensive tool evaluates methodological rigor across ten critical domains: selection bias (random sequence generation and baseline characteristics), performance bias (random housing and blinding), detection bias (random outcome assessment and blinding), attrition bias (incomplete outcome data), reporting bias (selective outcome reporting), and other potential sources of bias. Each criterion was rigorously appraised and categorized as "low risk," "high risk," or "unclear risk" based on the reported study methodologies, with particular attention to randomization procedures, blinding protocols, outcome assessment, and data completeness.

Quality assessment of included clinical trials was systematically evaluated using the Joanna Briggs Institute (JBI) Critical Appraisal Tool 16, a rigorously validated instrument specifically designed to assess risk of bias in clinical research, with particular applicability to correlational study designs. The evaluation criteria included ten items. Each item was evaluated as “Yes”, No”, “Unclear” or “Not Applicable”.

## Results

### Literature Search Results

**Figure 1:**
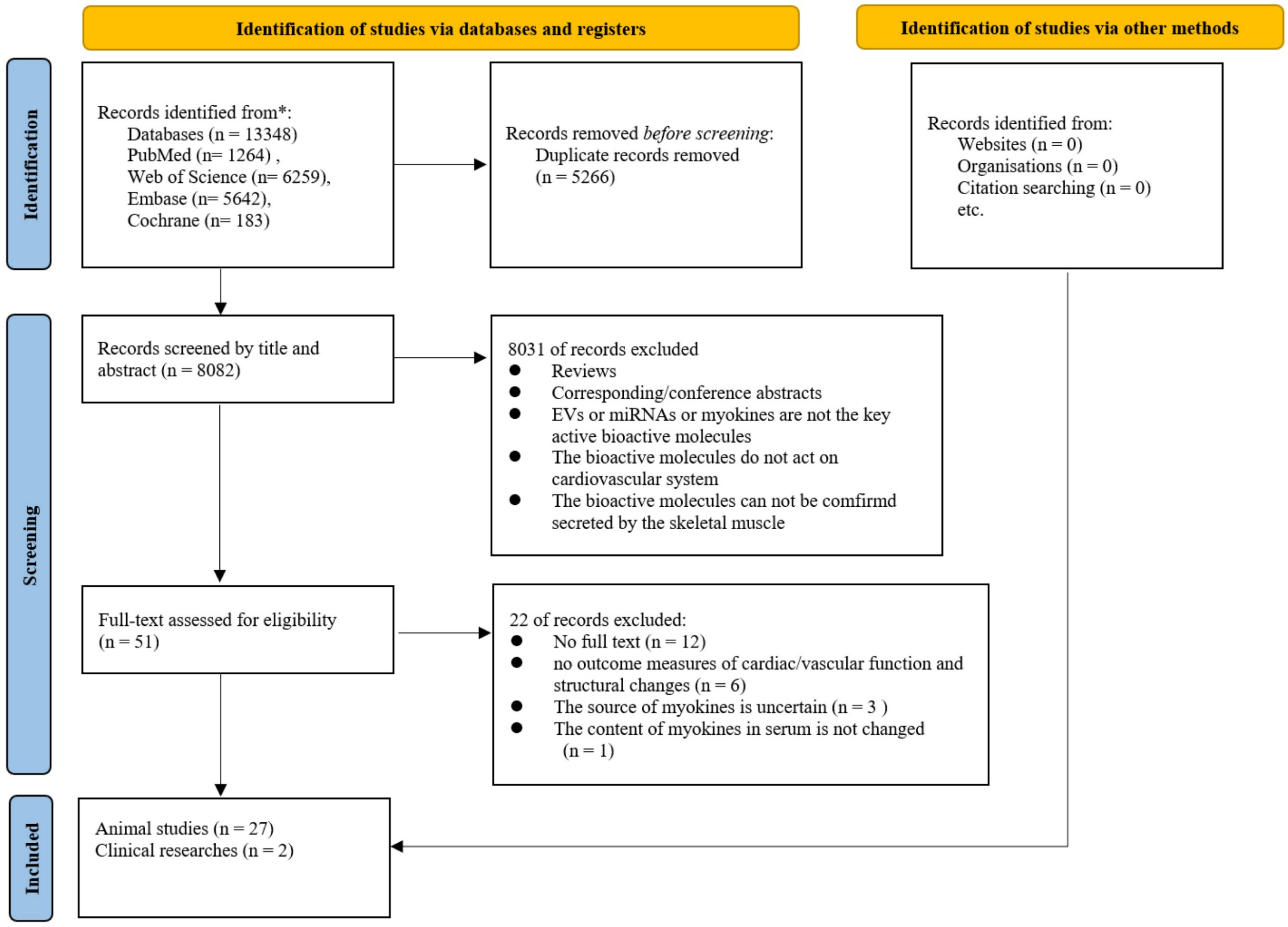
Flowchart of Literature Search and Screening.

The flowchart of the literature search and study selection process was presented in Figure 1. Based on the initial search criteria, we identified 13,348 records from four electronic databases. After removing 5,266 duplicate articles, we screened the remaining 8,082 records by title and abstract, excluding 8,081 articles. Following full-text evaluation of the remaining 51 articles, we excluded 22 studies. Finally, 29 articles 17-45 (2 clinical studies and 27 basic studies) were included in this study.

### Quality Assessment of Included Studies

The risk of bias assessment of the included animal experimental studies was presented in Supplement 2. According to SYRCLE's ROB tool evaluation, several methodological characteristics - including sequence generation, random outcome assessment, blinding procedures, and other potential bias sources - remained unclear. However, most studies demonstrated low risk concerning baseline characteristics, random housing, incomplete outcome data, and selective outcome reporting.

The JBI critical appraisal quality assessment results for the risk of bias and the method quality of the two included clinical studies were shown in Supplement 2. Both investigations followed standardized protocols for all the participants and were deemed methodologically reliable. These studies provided comprehensive reporting of participant demographics and clinical characteristics, clearly documented outcomes and follow-up results, and employed appropriate statistical analyses.

### Basic Characteristic of Selected Research

Two clinical studies and twenty-seven basic studies were included. The details of them were summarized in Table 1. The highest number of published articles were authored by researchers from Japan, followed by those from the United States and China. The selected studies primarily investigated the mechanisms and effects of skeletal muscle-derived bioactive molecules on using various animal models and cell cultures. These bioactive molecules originated from skeletal muscle tissues, C2C12 mouse myoblasts or recombinant proteins. The production methods of these molecules were categorized as: exogenous administration (n=19), endogenous stimulation (n=6), or both approaches (n=4). The exogenous methods involved stimulation/injection of recombinant proteins, transgenic animal models, adeno-associated virus (AAV)- mediated gene delivery and molecular mimics. The specific methods of endogenous stimulation included exercise, remote limb ischemia, or muscle wasting models. The main animal models employed comprised: five studies using ischemia-reperfusion (I/R) models, four studies with heart failure (HF) models, and one study each for vascular injury and atherosclerosis (AS) models. Among the twenty-seven basic studies, cellular experiments were performed in eleven studies and animal experiments were performed in twenty-four studies. Additionally, nine articles specifically investigated EVs.

### The Effects of Skeletal Muscle-derived EVs on the Heart and Blood Vessels

Nine articles investigating EVs were included. From a cargo composition perspective, three studies EV-containing proteins including vascular endothelial growth factor (VEGF), fibronectin type III domain-containing protein 5/irisin (FNDC5/irisin), and musclin; four studies reported EV-associated microRNAs including miR-206, miR-16-5p, and miR-126; while two studies did not provide clear descriptions of EV contents. Given the pivotal role of EV cargos in mediating cardiovascular effects, their biological functions were systematically categorized into myokine-related and miRNA-related mechanisms based on respective cargo compositions, as detailed in subsequent sections. Notably, two studies did not investigate EV cargo components. One study demonstrated that oxidative muscle-derived EVs exhibited superior pro-angiogenic effects compared to glycolytic muscle-derived EVs, manifested through significant enhancing of human umbilical vein endothelial cell (HUVEC) viability, proliferation, migration, tube formation and activation of the protein kinase B (Akt) /endothelial nitric oxide synthase (eNOS) signaling pathway. Another key study revealed that exercise-induced muscle EVs attenuated atherosclerotic plaque size and number in apolipoprotein E-deficient (ApoE^-/-^) mice, while using GW4869 (an EV secretion inhibitor) counteracted these cardioprotective effects.

### The Effects of Skeletal muscle-derived myokines on the Heart and Blood Vessels

Twenty-one studies investigated the effects of myokines on cardiac and vascular systems, with detailed findings presented in Table 2 and Supplement 3. The target organs or cells of myokines included the heart, cardiomyocytes, blood vessels and endothelial cells. Among them, there were fourteen studies targeting the heart or cardiomyocytes, and the main effects included improving cardiac function, alleviating myocardial fibrosis, alleviating cardiac injury and infarct size, inhibiting myocardium apoptosis and inflammatory response. There were thirteen articles targeting blood vessels or endothelial cells, some of which acted on both the heart and blood vessels. The primary effects on blood vessels included promoting angiogenesis and endothelial cell function.

Sixteen studies investigated myokines, including Follistatin-like 1 (FSTL1), musclin, myonectin, FNDC5/irisin, VEGF, IL-10, basic fibroblast growth factor (b-FGF), and acidic fibroblast growth factor (a-FGF), while five additional studies focused on skeletal muscle cell transplantation into the heart. Among them, FSTL1 was investigated in four studies. Three demonstrated its direct effects of HUVECs, promoting endothelial proliferation and migration and angiogenesis via activating vascular endothelial growth factor receptor 2 (VEGFR2)/Akt, Src/vascular endothelial cadherin (VE-Cadherin), phosphoinositide 3-kinase (PI3K)/AKT/eNOS, transforming growth factor beta 1 (TGFβ1)-Smad2/3 signaling pathways and up-regulating disco-interacting protein 2 homolog A (DIP2A) expression. Two of them used AAV to overexpress FSTL1. Another study found that skeletal muscle-specific FSTL1 knockout exacerbated percent luminal narrowing and neointimal thickening in response to vascular injury, while overexpression attenuated neointimal hyperplasia in vascular injury mice model, and suppressed proliferative and migratory activities of human aortic smooth muscle cells (HASMCs) through activation adenosine monophosphate-activated protein kinase (AMPK) signaling pathway. However, one study found that AAV-FSTL1 overexpression in skeletal muscle had no significant effect on promoting vascularization and improving cardiac function and remodeling in myocardial infarction (MI) rat model. Skeletal muscle-specific musclin overexpression in one study demonstrated its inhibition of cardiac fibrosis and dysfunction and improvement of ventricular contractility during chronic pressure overload via competitively bonding to the natriuretic peptide receptors C (NPRC) and increasing the C-type natriuretic peptide (CNP)/natriuretic peptide receptor B (NPR-B) signaling, while musclin knockout aggravated the advancement of HF. There were two studies focused on FNDC5/irisin. One literature showed the skeletal muscle-specific FNDC5/irisin knockout exacerbated vascular stiffness and senescence in aged or AngII-induced mice model by activating the DnaJ/ heat shock protein 40 (Hsp40) chaperone system to stabilize Sirtuin 6 (SIRT6). Another clinical trial found that FNDC5/irisin was positively related to aerobic performance in the HF patients. There were four studies related to VEGF, all of which described that VEGF promoted angiogenesis. Three of them found that it improved cardiac function and alleviated myocardial injury and fibrosis, thereby protecting the heart in cardiac injury models. Two studies respectively employed the methods of injecting VEGF protein into muscle or injecting skeletal muscle cells overexpressing VEGF into the heart. One study revealed that skeletal muscle-specific myonectin knockout aggravated myocardial ischemic injury by suppressing apoptosis and inflammatory reactions, whereas its overexpression attenuated myocyte apoptosis by promoting the cyclic adenosine monophosphate (cAMP)-dependent activation of AKT in myocardial ischemia reperfusion injury (MIRI) mice. There were two studies on the effects of IL-10. One study found that IL-10 reduced myocardial infarction size and improved cardiac function through the PI3K/Akt signaling pathway in MIRI rat. Another study found it prolonged graft survival and reduced histologic rejection by reducing neutrophil content in the allotransplanted heart model rat. Two studies related to b-FGF and a-FGF. Both showed that b-FGF increased cardiac angiogenesis by intramuscular injection. But skeletal muscle derived a-FGF did not significantly increase vascularization in infarcted heart. The remaining five studies evaluated skeletal muscle cell transplantation strategies, including myoblasts or non-myogenic cells sheets, triple-layer skeletal muscle (SkM) sheets, myogenic differentiation factor knockout (MyoD^-/-^) and wild-type myoblasts, skeletal myoblasts (SMBs) or mononuclear bone marrow cells (BMCs) and skeletal muscle and mesenchymal stem cells. All of them showed that they improved cardiac function in MI animals by inhibiting apoptosis or inflammation response. And three studies demonstrated angiogenesis promotion. One study found that employing cell sheets technology reduced the incidence of arrhythmia after transplantation.

Regarding the source of myokines, among twenty-one articles, individual studies had documented that FSTL1, FNDC5/irisin, musclin and myonectin were confirmed to originate exclusively from skeletal muscle through skeletal muscle-specific AAV interventions or transgenic mouse models, establishing their skeletal muscle origin and subsequent effects on the heart and blood vessels. Fourteen studies employed intramuscular injection of recombinant protein, non-muscle-specific AAV or plasmid, or skeletal muscle cells, including VEGF, b-FGF, a-FGF, FSTL1, IL-10 and various skeletal muscle cells. The remaining three studies employed either exercise or direct cellular interventions.

### The Effects of Skeletal Muscle-Derived miRNAs on the Heart and Blood Vessels

Six studies investigated the effects of skeletal muscle-derived miRNAs on cardiovascular systems, as detailed in Table 3 and Supplement 4. Different miRNAs played different roles. There were two studies targeting the heart or cardiomyocytes to protect or injury the heart. There were four articles targeting blood vessels or endothelial cells to promote angiogenesis and endothelial cell function. These skeletal muscle-derived miRNAs mainly included miRNA-206, miRNA-16-5p, miRNA-26a, and miRNA-126. Among them, two studies demonstrated that miRNA-206 improved endothelial cell function and promoted angiogenesis via reactive oxygen species (ROS)/ nuclear factor kappa B (NF-κB) signaling pathway, but not VEGF-dependent signaling pathway. They respectively utilized miRNA-206 mimics and EV-miRNA-206 in HUVECs. One study mainly showed that elevated miRNA-16-5p from atrophic muscle disturbed cardiac repair by modulating the apoptosis and inhibiting autophagy in MIRI mice. A single study highlighted that exo-miRNA-26a, containing muscle surface peptides, attenuated cardiac fibrosis and improved cardiac function through mediating forkhead box O1 (FOXO1) downregulation in chronic kidney disease (CKD) mice. Two studies indicated that miRNA-126 promoted endothelial cells (ECs) proliferation and angiogenesis via the PI3K/Akt signaling pathway. They respectively employed exercise or miRNA-126-5p agomir to increase the expression of miRNA-126-5p. Regarding the source of miRNAs, one study used muscle peptide-labeled exosomes carrying miRNA-26a, three employed miRNA plasmids/mimics (for miRNA-206, -16-5p, and -126), and two utilized exercise or direct cellular interventions.

### The Effects of EVs and Bioactive Molecules from Skeletal Muscle on Heart

Seventeen studies investigated the effects of skeletal muscle-derived EVs, myokines, miRNAs, and cells on the heart. These details were shown in Table 4. This article summarized different roles of the bioactive molecules and skeletal muscle cells on the heart. Twelve studies each reported that musclin, VEGF, IL-10, myonectin, myoblasts, skeletal muscle cells and exo-miRNA-26a enhanced cardiac function. Among them, musclin and IL-10 performed improvement of cardiomyocyte contractility. Five studies respectively reported that musclin, VEGF, myoblasts and exo-miRNA-26a decreased cardiac fibrosis. Six studies respectively showed that myonectin, IL-10, VEGF, skeletal muscle cells and myoblasts reduced cardiac injury or myocardial infarct size in animal models of I/R injury. Three studies each demonstrated that myonectin, myoblasts and VEGF protected the heart via suppressing myocardial apoptosis. Three studies respectively showed that myonectin, skeletal muscle cells and myoblasts inhibited inflammatory responses. One clinical study reported that FNDC5/irisin was associated with improving aerobic performance in the HF patients. One article showed that skeletal muscle-derived VEGF attenuated progression of HF by promoting myocardial regeneration and cardiac repair. One study demonstrated that EV-miRNA-16-5p dysregulated the balance between apoptosis and autophagy under oxidative stress; specifically, promoting cardiomyocyte apoptosis and decreasing autophagy.

### The Effects of EVs and Bioactive Molecules from Skeletal Muscle on Blood Vessels

Twenty studies reported on the roles of skeletal muscle-derived EVs, bioactive molecules, and cells on the blood vessels. The detailed results presented in Table 5. Seventeen articles each demonstrated that FSTL1, EV-miRNA-206, VEGF, myoblasts, b-FGF, skeletal muscle cells and miRNA-126 promoted angiogenesis and capillary growth. Nine studies confirmed that FSTL1, skeletal muscle-derived EVs and MyoD^-/-^ and wild-type myoblasts, miRNA-126-5p and EV-miRNA-206 improved endothelial cell function, including its proliferation, migration and tube formation. One study demonstrated that FSTL1 alleviated neointimal hyperplasia by inhibiting the proliferation and migration of smooth muscle cells. One study demonstrated that skeletal muscle-derived EVs alleviated atherosclerosis though reducing the plaque size and number in aorta in the ApoE^-/-^ mice, whereas exosomal inhibitor GW4869 counteracted the protective effects. One study showed that EV-irisin protected against vascular stiffness and senescence by increasing the stability of SIRT6.

## Discussion

Each organ did not function independently in human body. There existed the information exchange between them, which was called as interorgan communication 46. It was essential for maintaining normal function and dynamic balance in human body. Interorgan communication was coordinated through a complex regulatory network of secretory factors 47 and adjusted response to environmental changes 46. The bioactive molecules released by skeletal muscle played a dominant role because it was described as the body's largest endocrine organ. These EVs, myokines and miRNAs mediated the communication with the neighboring cells and distant organs by entering the extracellular fluid and blood circulation.

The systematic review of 2 clinical studies and 27 basic studies demonstrated that the skeletal muscle communicated with the heart and blood vessels by EVs, myokines, and miRNAs. Through summarizing the effects of skeletal muscular-derived myokines on the heart and blood vessels, our studies found that most of them improved cardiac function, reduced cardiac fibrosis and injury, inhibited apoptosis and inflammation, enhanced angiogenesis and endothelial cell function by targeting the various critical signaling pathways. Moreover, in the present studies, some investigated the protective effects of various bioactive molecules on the same animal model, for instance, VEGF, IL-10, myonectin and FSTL1 acting positive effects in the I/R model. However, the same bioactive molecule could elicit similar or distinct effects in different models, such as VEGF. Therefore, it was very valuable to analyze the specific roles of different myokines and miRNAs on the heart and blood vessels in same or different disease models.

### The Effects of EVs on the Heart and Blood Vessels

This study concluded that skeletal muscle-derived EVs promoted angiogenesis and alleviated atherosclerosis 43, 44. EVs were carriers of nucleic acids, proteins, lipids and other molecules from the parent cells. It protected these contents from hydrolysis. They mediated intercellular communication in a paracrine manner, or interorgan communication by entering the blood circulation and reaching the distant organs. Besides, EVs demonstrated exceptional stability, biocompatibility, wide biodistribution, and minimal immunogenicity. Thus, in recent years, EVs have been used to research intercellular and interorgan communication 48, 49, their value as novel clinical biomarkers 50, or as bioengineered vehicles for delivering genetic material or drugs 51, 52. EVs were the main vehicles of skeletal muscle-derived bioactive molecules, though some studies did not focus on them, such as FSTL1 53, IL-10 54, FNDC5/irisin 55, VEGF 56, FGF 57, miRNA-126 58. However, these factors were the key to function. Their effects on the heart and blood vessels needed to be further investigated.

### The Effects of Myokines on the Heart and Blood Vessels

Myokines were cytokines mainly produced and secreted by the skeletal muscle. They target and exert distant organs in an endocrine manner. Studies had identified hundreds of myokines 13, including inflammatory cytokines, growth factors, metabolic-related factors, and other factors. This study focused on the effects of myokines derived from the skeletal muscle on the heart and blood vessels.

### Inflammatory Factors as Myokines

Inflammatory factors, such as IL-6 10, IL-7 59, IL-10, IL-15 60, also acted as myokines. In this study, IL-10 was the main concern. It, as a well-known anti-inflammatory cytokine, suppressed activation and effector functions of the T cells, monocytes, and macrophages 61. One included study 34 showed that intramuscular injection of IL-10 increased and protected the myocardium, reduced infarct size in the I/R model mice. Studies demonstrated enhancing the levels of IL-10 significantly improved left ventricular function, reduced infarct size, alleviated ventricular wall injury, and increased capillary density by activating signal transducer and activator of transcription 3 (STAT3) in the MI model mice 62-64. Furthermore, another included study 38 showed that IL-10 suppressed of rejection of heart allograft transplantation by employing intramuscular administration of IL-10 plasmid. This directly substantiated the cardioprotective role of IL-10 through the communication from skeletal muscle to the heart. Generally, chronic rejection significantly affected the long-term survival of heart transplant patients. Studies demonstrated that IL-10 played a key role in the macrophage-mediated post-transplantation immune response. Researchers found that IL-10 alleviated chronic graft rejection by increasing macrophage M2 polarization 65, inhibiting T lymphocyte infiltration and cytotoxicity 66. These results demonstrated that IL-10 protected against the myocardium injury and suppressed rejection of allogeneic heart transplantation partly through its anti-inflammatory role.

### Growth Factors as Myokines

Skeletal muscle also produced and secreted growth factors, such as FGF 67, 68, VEGF^69^, ^70^. There were many subtypes of FGF, frequently, a-FGF and b-FGF. They activated intracellular signal transduction pathways by binding to the fibroblast growth factor receptor (FGFR) on the target cell surface, thereby promoting cellular proliferation, differentiation, and migration. Two included studies 39, 40 showed that intramuscular injection of b-FGF significantly increased angiogenesis in the rat heart and gastrocnemius muscles. B-FGF was a potent pro-angiogenic factor and acted in synergy with VEGF in various tissues 71, 72. Therefore, intramuscular injection of b-FGF promoted angiogenesis in the heart through interorgan communication. Four included studies 22, 32, 35, 42 suggested that the main effects of VEGF were promoting angiogenesis and improving cardiac function and attenuating myocardial injury and fibrosis. Two of them 35, 42 respectively employed the methods of injecting VEGF protein into muscle or injecting skeletal muscle cells overexpressing VEGF into the heart. The family of VEGF included several subtypes, mainly involving VEGF-A, VEGF-B, VEGF-C and VEGF-D. Studies showed that VEGF-A/B inhibited cardiomyocyte apoptosis and activated the expression of genes associated with myocardial contraction and metabolism 73. Designed protein VEGF-CC152S accelerated myocardial lymphangiogenesis, alleviated cardiac inflammation, fibrosis, and dysfunction, and reduced ventricular remodeling after MI 74. Related studies showed that VEGF-A/C reduced fiber scar area by effectively promoting collateral circulation, reducing edema, and improving cardiac function after MI 75. Different phenotypes of VEGF might have different effects on myocardium. But in general, they all protected the heart after MI.

### Metabolism-Related Myokines

Irisin and musclin mainly derived from skeletal muscle and were important myokines. They were originally found to exerted an important role in metabolism 76, 77. One included study 20 firstly found that irisin alleviated vascular stiffness and inflammation by stabilizing SIRT6 in the vascular injury models. Studies reported that SIRT6 prevented vascular smooth muscle cells (VSMCs) from aging and maintained vasodilation function by blocking the expression of NF-κB-dependent receptor 78, 79. And irisin suppressed vascular aging by inhibiting inflammation and oxidative stress 80. Other studies demonstrated that skeletal muscle-derived irisin played an anti-aging role in multiple target organs 81. Another included study showed that irisin levels 33 showed positive correlation with the aerobic capacity of patients with HF after exercise. Aerobic capacity was affected by cardiac output, myocardial energy metabolism, as well as the functions of the skeletal muscle and the diaphragm. Irisin increased the cardiac output of zebrafish 82 and protected mitochondria and improved myocardial energy metabolism 83. Irisin enhanced diaphragm function by increasing the levels of phospho-AMPK (pAMPK) 84. Furthermore, muscle loss was associated with reduced levels of irisin protein 85. Therefore, irisin protected vascular function through its anti-aging, anti-inflammation and anti-oxidative stress effects, and improved aerobic capacity by enhancing cardiac output, optimizing myocardial energy metabolism, and improving muscle function. One included study showed that musclin 19 inhibited myocardial fibrosis and increased myocardial contractility and accelerated HF progression via competitively bonding to the NPRC and increasing the CNP/NPRB signaling pathway in the HF model mice induced by transverse aortic constriction (TAC). In this study, using skeletal muscle-specific knockout of musclin and transgenic mice rendered the role of musclin in mediating skeletal muscle-heart communication more credible. Researches have proved that the partial amino acid sequences of musclin and natriuretic peptides (NPs) were homologous and it was an endogenous ligand of NPRC, but it did not demonstrate natriuretic peptide activity. Moreover, it entered blood circulation and acted on distant organs 86. NPRC was natriuretic peptide clearance receptor. So musclin protected the myocardium by competitively binding to NPRC. It suppressed development of HF by alleviating myocardial fibrosis 87, reducing myocardial infarction size and improving myocardial mitochondrial homeostasis 88. Musclin was derived mainly from skeletal muscle, and it protected the heart via the natriuretic peptide signaling.

### Other Myokines

FSTL1 and myonectin were important myokines. There were four studies about FSTL1 18, 21, 31, 36 in this study. They reported that the main roles of FSTL1 were promoting endothelial function and angiogenesis in different cell and animal models. Moreover, one of them 31 utilized skeletal muscle-specific FSTL1 transgenic mice model of vascular injury to confirm that FSTL1 reduced intimal hyperplasia. Previous studies have confirmed that FSTL1 promoted synthesis of angiogenic proteins during ischemic stress after MI by activating TGFβ-Smad2/3 89 and AMPK and mediating the production of nitric oxide (NO) 90. Epicardial injection of FSTL1 increased myocardial vascularization in the infarct margin area 91. Therefore, the role of FSTL1 in improving intimal hyperplasia and promoting vascular regeneration was evident and confirmed in vascular injury models. Myonectin, also known as C1q/tumor necrosis factor (TNF)-associated protein 15/erythroferrone, was a cardioprotective factor 92. According to the included studies, myonectin 28 reduced infarct size and inhibited cardiomyocyte apoptosis and macrophage inflammatory response in transgenic mice with skeletal muscle-specific overexpression of myonectin after MIRI. It proved that skeletal muscle-derived myonectin protected the heart trough circulation. Studies also have found that myonectin alleviated ventricular remodeling, myocardial fibrosis and dysfunction in the TAC mice 93, 94. At present, myonectin was recognized a novel cardioprotective factor.

However, the results of two included studies were not inconsistent with previous studies. They respectively showed that a-FGF and FSTL1 did not significantly improve damaged heart vascularization. However, several studies had shown that a-FGF also played a role in angiogenesis. They reported that local injections of a-FGF into the heart of MI model pigs promoted angiogenesis in the heart infarcted areas 95, 96. Possible reasons for this difference could be categorized into three: (1) they did not significantly increase in the myocardium; (2) blood perfusion was reduced in the infarct area; (3) they failed to reach the infarct area or exert their effects through the skeletal muscle-heart. Finally, the content of them in the infarct area was not sufficient to promote angiogenesis. Thus, their function in vascular regeneration following MI awaited further exploration through skeletal muscle-heart communication.

### The Effect of MiRNAs on the Heart and Blood Vessels

MiRNAs were small non-coding RNAs that regulated gene expression in cells and tissues. They were usually carried by the EVs and participated in interorgan communication to activating or suppressing downstream signaling pathways by modulating target gene expression of the recipient organs or cells. Therefore, they might be used as potential therapeutic target for various human diseases, including CVDs 97.

In included study, exosomes, containing muscle surface peptides, loaded with miRNA-26a were injected into the muscle. Furtherly, skeletal muscle-derived miRNA-26a 26 improved heart function and reduced myocardial fibrosis in CKD mice. Related studies showed that miRNA-26a not only reduced myocardial fibrosis, regulated autophagy 98 and reduced type I collagen 99, but also inhibited myocardial cell damage and alleviated myocardial fibrosis in the diabetic cardiomyopathy mice 100. Therefore, miRNA-26a played a positive role in the heart. In two included studies, the expression of miRNA-126 increased following the transfection of miRNA-126 into muscle tissue or exercise. And miRNA-126 25, 29 promoted endothelial cell proliferation and migration, and increased angiogenesis through the communication from skeletal muscle to the heart. MiRNA-126 was a major physiological and pathological regulator of angiogenesis 101. It regulated endothelial function by inhibiting endothelial cell apoptosis and promoted endothelium repair after injury 102, 103. Therefore, miRNA-126 played a protective role in the blood vessels and endothelial cells. Exosomal miRNA-206 17, 27 promoted angiogenesis by acting on endothelial cells. However, the underlying mechanisms were not clear and required further investigations. In included study, miRNA-16-5p 23 played a negative regulatory role by reducing autophagy, promoting cardiomyocyte apoptosis and inhibiting cardiac repair in the MIRI model mice after muscular atrophy. Related studies showed that reduced expression of miRNA-16-5p promoted hypertrophy of cardiomyocytes in the newborn rats 104 but induced angiogenesis in the hearts of obese rats by increasing VEGF expression 105. Therefore, the underlying mechanisms of miRNA-16-5p on heart function and heart diseases required further in-depth analysis.

In summary, the degree of confidence in the role of skeletal muscle-derived myokines and miRNAs in the heart or blood vessels was inconsistent. Among them, IL-10, b-FGF, VEGF, irisin, musclin, myonectin and exo-miRNA26a, and miRNA-126 had definite protective effects on the heart or blood vessels. In particular, irisin, musclin and myonectin used skeletal muscle specific overexpression and demonstrated that they were involved in communication from skeletal muscle to the heart or blood vessels and had protective effects on them. FSTL1 derived from skeletal muscle had a definite protective effect on blood vessels. However, whether it and a-FGF mediated communication between skeletal muscle and heart was not clear. Moreover, researches on miRNA-206 and miRNA-16-5p were less and immature and not enough to prove their roles in included studies.

### Different Targets and Functions of Various Myokines and miRNAs

Different myokines and miRNAs acted on the same pathological processes of the heart or blood vessels, while the same myokines and miRNAs could also influence different pathological processes. This phenomenon may be related to the broad range of molecular targets affected by myokines and miRNAs 106.

### Limitations

This study still had a few limitations. Firstly, the included literature exhibited significant heterogeneity. Different myokines were capable of regulating various pathological processes in the same animal model, while the same myokine could influence similar or different pathological mechanisms across different models. Consequently, it was a challenge to draw consistent conclusions regarding the effects of some bioactive molecules derived from skeletal muscle on the heart and blood vessels. Secondly, a few studies on EVs did not perform tracking experiments to confirm EV transfer from skeletal muscle to the heart or blood vessels. Furthermore, none of the studies used specific fluorescent labeling to trace myocyte-derived myokines or miRNAs for subsequent observation in cardiac or vascular tissues. These issues need to be addressed in future studies.

## Conclusion

This article systematically reviewed the included literature and analyzed the mechanisms by which skeletal muscle-derived EVs, myokines and miRNAs regulated cardiac and vascular function and structure, based on 2 clinical studies and 27 basic studies. Three main findings are summarized: First, there exists interorgan communication from skeletal muscle to the heart and blood vessels via myokines and miRNAs, some of which are carried and transported by EVs. Second, most of the myokines and miRNAs demonstrate beneficial effects on the heart and blood vessels. In the heart, they enhance cardiac function, reduce cardiac fibrosis and injury, and inhibit cardiomyocyte apoptosis and inflammation. In the blood vessels, they promote angiogenesis and capillary growth, improve endothelial cell function, protect against vascular stiffness and attenuate atherosclerosis and neointimal hyperplasia. Third, research on the role of skeletal muscle-derived myokines and miRNAs in the heart or blood vessels is uneven. Among these factors, IL-10, b-FGF, VEGF, irisin, musclin, myonectin, exo-miRNA26a, and miRNA-126 have been confirmed to exert protective effects on the heart or blood vessels through various physiological and pathological processes. Specifically, studies using skeletal muscle-specific overexpression demonstrate that irisin, musclin, and myonectin mediated communication from skeletal muscle to the heart or blood vessels, contributing to their protection. Additionally, skeletal muscle-derived FSTL1 has a clear protective effect on blood vessels; however, it remains unclear whether FSTL1 and a-FGF are involved in skeletal muscle-heart crosstalk. Furthermore, research on miRNA-206 and miRNA-16-5p remains limited and inconclusive, with insufficient evidence to support their roles in the included studies.

Overall, these results elucidate the mechanisms by which skeletal muscle-derived EVs, myokines and miRNAs improve cardiac and vascular function and structure through interorgan communication, as well as how exercise may prevent and ameliorate CVDs by bioactive molecules released by skeletal muscle. IL-10, FSTL1, b-FGF, VEGF, irisin, musclin, myonectin, exo-miRNA26a, and miRNA-126 provide new targets for further mechanistic and clinical research on communication from skeletal muscle to the heart and blood vessels. Meanwhile, these findings also provide a possibility for developing skeletal muscle-based therapies for CVDs.

## Future Perspectives

The roles of myokines and miRNAs are complex, and more in-depth and comprehensive mechanism studies are needed in the future.

## Figures and Tables

**Table 1:** The Basic Characteristics Information of All Studies

Author, year	Journal	Country	Sources of bioactive molecules	Ways of producing bioactive molecules	Objects of study	Models/Diseases	Carried by EVs
Kargl, 2023 44	scientific reports	America	oxidative and glycolytic muscle tissue in mice	exogenous (bioactive molecules stimulation)	cells	N/A	Yes
Wang, 2023 43	Aging and Disease	China	muscle	endogenous (exercise)	animals	atherosclerosis	Yes
Li, 2023 18	American journal of physiology. Cell physiology	China	recombination proteins	exogenous (bioactive molecules stimulation)	cells	N/A	No
Xi, 2022 21	Journal Sport and Health Science	China	skeletal muscle	endogenous and exogenous (exercise and AAV)	animals	MI	No
			recombination proteins	exogenous (bioactive molecules stimulation)	cells	N/A	
Malgorzata, 2022 19	Nature Communication	Germany	skeletal muscle	exogenous (AAV)	animals	HF	No
Hiroya, 2022 17	circulation journal	Japan	serum of Akt1 transgenic mice	exogenous (transgenic mice)	animals	skeletal muscle growth model	Yes
			C2C12 mouse myoblasts	exogenous (bioactive molecules stimulation)	cells	Akt overexpression	
Chi, 2022 25	European Heart Journal	China	skeletal muscle	endogenous and exogenous (AAV and exercise)	animals	vascular ageing	Yes
			recombination proteins	exogenous (bioactive molecules stimulation)	cells	vascular ageing	
Rubens, 2021 22	Frontiers in Physiology	America	skeletal muscle	endogenous (remote hind-limb ischemia)	animals	CHF model	Yes
Taiki, 2020 23	European Heart Journal	Japan	the atrophic limbs sarcopenic mice	endogenous and exogenous (TS and miRNA-16-5p mimics)	animals	MIRI mouse model	Yes
			miRNA‑16‑5p mimics		cells	N/A	No
Hiroko, 2020 24	Stem Cell Research Therapy	Japan	skeletal muscle	exogenous (cell transplantation)	animals	MI	No
Chen, 2020 25	Aging	China	temporal muscle	exogenous (agonist stimulation)	animals	CCI model	No
			microRNA-126-5p mimics		cells	N/A	No
Nie, 2019 27	Experimental Physiology	America	C2C12 myotube	exogenous (exogenous molecules injection)	cells	hypoxia	Yes
Wang, 2019 26	Theranostics	China	muscle satellite cells	exogenous (bioactive molecules injection)	animals	chronic kidney disease	Yes
Naoya, 2018 28	Circulation Research	Japan	skeletal muscle (soleus)	endogenous (exercise)	animals	MIRI	No
			recombinant proteins	exogenous (bioactive molecules stimulation)	cells	hypoxia/reoxygenation	
João Lucas, 2017 29	Oxidative Medicine and Cellular Longevity	Brazil	skeletal muscle	endogenous (exercise)	animals	obesity	No
Megumi, 2014 31	Cardiovascular Research	Japan	skeletal muscle	exogenous (transgenic mice)	animals	vascular injury	No
			recombinant proteins	exogenous (bioactive molecules stimulation)	cells	N/A	
Birgitte, 2013 32	Faseb Journal	Denmark	skeletal muscle fibers	endogenous (exercise)	subjects	N/A	Yes
Yutaka, 2013 30	Cell Medicine	Japan	N/A	exogenous (cells transplantation)	animals	MI	No
Cai, 2012 34	Basic Research in Cardiology	America	gastrocnemius muscle	endogenous (Remote ischemic preconditioning)	animals	MIRI	No
Stewart, 2012 33	Circulation-Heart Failure	Boston	skeletal muscle	endogenous (exercise)	subjects	HF	No
Yasuhiro, 2012 45	Plos one	Japan	skeletal muscle	exogenous (cells transplantation)	animals	MI	No
David, 2009 35	American Journal of Physiology-Regulatory Integrative and Comparative Physiology	America	recombinant proteins	exogenous (bioactive molecules injection)	animals	HF	No
Noriyuki, 2008 36	Journal of Biological Chemistry	Boston	gastrocnemius muscle	exogenous (AAV)	animals	hind limb ischemia model	No
					cells	N/A	
Steven, 2008 37	circulation	America	N/A	exogenous (cells transplantation)	animals	post-MI CHF	No
Efthimiadou, 2006 39	British Journal Sports Medicine	Greece	recombinant protein	exogenous (bioactive molecules injection)	animals	tenotomy model	No
Carvalho, 2006 41	Transplantation Proceedings	Brazil	N/A	exogenous (cells transplantation)	animals	MI	No
						CD	
Reza, 2006 38	Journal of Gene Medicine	Switzerland	left tibialis anterior muscle	exogenous (bioactive molecules injection)	animals	heterotopic heart transplantation	No
Claudia, 2006 42	International Journal of Cardiology	Brazil	skeletal myoblasts transfected withVEGF	exogenous (cells transplantation)	animals	MIRI	No
Efthimiadou, 2006 40	Journal of Sports Science	Greece	recombinant protein	exogenous (bioactive molecules injection)	animals	N/A	No

AAV: adeno-associated virus; AKT: protein kinase B; CCI: chronic cerebral ischemia; CD: chagas's disease; CHF: chronic heart failure; EVs: extracellular vesicles; HF: heart failure; HUVECs: human umbilical vein endothelial cells; MI: myocardial infarction; MIRI: myocardial ischemia reperfusion injury; miRNA: microRNA; NRVMs: neonatal rat ventricular myocytes; N/A: Not Applicable; TS: tail suspension; VEGF: vascular endothelial growth factor.

**Table 2:** The Effects of Skeletal muscle-derived Myokines on the Heart and Blood Vessels

Author, year	Sources of Myokines	Myokines	Models/ Diseases (Methods)	Targeted organs/ cells	Effects on heart/blood vessels
Li, 2023 18	exogenous FSTL1	FSTL1	N/A	HUVECs	rhFSTL1 stimulated HUVECs: cell proliferation and migration↑; angiogenesis↑; intercellular adhesion junctions↓
Xi, 2022 21	skeletal muscle	FSTL1	MI rat model (LAD ligation)	heart and blood vessels	AAV-FSTL1 overexpression regulated heart and blood vessels: vascularization of the infarcted myocardium→; cardiac function→; cardiac remodeling→
	rhFSTL1		N/A	HUVECs	RhFSTL1 regulated HUVECs function: ECs proliferation↑;
Malgorzata, 2022 19	skeletal muscle	musclin	HF mice model (TAC)	heart	Skeletal muscle-derived musclin showed the effects in the heart: cardiac fibrosis↓; cardiac function↑; HF progression↓; ventricular contractility↑
Chi, 2022 20	skeletal muscle	EV-FNDC5/irisin	vascular ageing mice model (Ang II treatment)	artery	Skeletal muscle-specific FNDC5 overxepression played a role in arteries: vascular stiffness and senescence↓; inflammatory responses↓
	exogenous FNDC5/irisin		vascular ageing (Ang II treatment)	VSMCs	Exogenous irisin added in AngII-induced VSMCs: vascular stiffness and senescence↓
Rubens, 2021 22	skeletal muscle	EV- VEGF	CHF mice model (AV fistula)	heart and blood vessels	Skeletal muscle-derived exosomes exerted the effects in the heart and blood vessels: 1. myocardial fibrosis↓; cardiac function↑ 2. endothelial function↑; angiogenesis↑
Hiroko, 2020 24	skeletal muscle	myoblasts or non-myogenic cells sheets	MI rat model	heart	Cell sheets consisting of myoblasts and non-myoblasts transplanted in heart: cardiac function↑; myocardial fibrosis↓; infarct size→; angiogenesis↑
Naoya, 2018 28	soleus	myonectin	MIRI murine model	heart	Skeletal muscle-specific myonectin overexpression exerted the effects in the heart: cardiac function↑; ischemic injury↓; apoptosis↓; inflammatory responses↓
	recombinant myo nectin protein		H/R	myocytes	Myonectin treatment in myocytes: myocyte apoptosis↓
Megumi, 2014 31	skeletal muscle	FSTL1	vascular injury mice model (wire injury operation)	blood vessels	Skeletal muscle-specific FSTL1 overexpression played a role in the blood vessels: percent luminal narrowing↓; neointimal thickening↓; inflammatory responses↓
	rhFSTL1		N/A	HASMCs	Recombinant human FSTL1 played a role in HASMCs: HASMCs proliferation and migratory↓
Birgitte, 2013 32	endogenous	EV-VEGF	N/A	capillaries	Capillary growth↑
Yutaka, 2013 30	N/A	triple-layer SkM sheets	MI porcine model (LAD occlusion)	heart	SkM Sheet transplanted in heart: cardiac function↑; ventricular arrhythmia vulnerability↓; local injury↓; inflammation responses↓
Zheqing, 2012 34	gastrocnemius muscle	IL-10	MIRI mice model	heart	RhIL-10 injected in IL-10 KO and RIPC mice: cardiac function↑; infarct size↓; cardiac contractility↑
Stewart, 2012 33	skeletal muscle	FNDC5	HF patients	heart	Skeletal muscle-derived FNDC5 played roles in HF patients: positively related to aerobic performance
Yasuhiro, 2012 45	skeletal muscle	MyoD-/- and wild-type myoblasts	MI rat model (LAD ligation)	heart	Wild-type and MyoD^-/-^ myoblasts injected into infarcted mouse heart: cardiac function↑; infarct size↓; apoptosis↓; survival↑; angiogenesis↑
David, 2009 35	rhVEGF	VEGF	HF (Bio-TO2 male hamsters)	heart and blood vessels	VEGF intramuscularly injected in the TO2 hamster: ventricular function↑; apoptosis↓; myocardial injury↓; myocardial fibrosis↓; cardiomyogenesis↑; angiogenesis↑
Noriyuki, 2008 36	gastrocnemius muscle	FSTL1	hind limb ischemia murine model	blood vessels	AAV-FSTL1 overexpression played a role in mice: revascularization↑
	N/A		N/A	HUVECs	AAV-FSTL1 overexpression played a role in HUVECs: endothelial cell function↑; apoptosis↓; survival↑
Steven, 2008 37	N/A	SMBs or mononuclear BMCs	post-MI CHF rats (LAD occlusion)	heart	SMBs or mononuclear BMCs transplanted in the heart: cardiac function↑; inflammatory responses↓; gap junctions between grafts and native cardiomyocytes↓
Efthimiadou, 2006 39	injected in the right gastrocnemius muscle	b-FGF	tenotomy rat model	blood vessels	B-FGF injection into the gastrocnemius muscle exerted a role in blood vessels: angiogenesis↑
Carvalho,2006 41	N/A	skeletal muscle and mesenchymal stem cells	MI rat model (LAD occlusion)	heart and blood vessels	Skeletal muscle and mesenchymal stem cells transplanted in the heart: cardiac function↑; myotube formation↑; angiogenesis↑
			CD rat model		Skeletal muscle and mesenchymal stem cells transplanted in the heart: cardiac function↑; angiogenesis↑
Reza, 2006 38	injected in lefttibialis anterior muscle	IL-10	heterotopic heart transplantation rats	heart	Injection of IL-10 plasmid into the tibialis anterior muscle played a role in the heart: graft survival↑; histologic rejection↓; neutrophil content↓
Claudia, 2006 42	skeletal myoblasts transfected withVEGF	myoblasts with overexpression VEGF	MIRI rat model (LAD occlusion)	heart and capillary	Injection of skeletal myoblasts with overexpression VEGF into the MIRI rat heart: infarct size↓; cardiac damage↓; angiogenesis↑
Anna, 2006 40	injected intramuscularly in the right gastrocnemius	b-FGF, a-FGF	Rats	heart and blood vessels	Intramuscular injection of b-FGF and a-FGF into the gastrocnemius muscle resulted in the effects on cardiac vessels: b-FGF: angiogenesis↑ a-FGF: angiogenesis→

AAV: adeno-associated Virus; Ang II: angiotensin I; AV: aorta-vena-cava; a-FGF: acidic- fibroblast growth factor; BMCs: bone marrow cells; b-FGF: basic-fibroblast growth factor; CD: chagas's disease; CHF: chronic heart failure; EV: extracellular vesicle; FNDC5: fibronectin type-III domain-containing protein 5; FSTL1: follistatin-like 1; HASMCs: human aortic smooth muscle cells; HF: heart failure; HUVECs: human umbilical vein endothelial cells; H/R: hypoxia/reoxygenation; IL-10: interleukin-10; KO: knockout; LAD: left anterior descending; MI: myocardial infarction; MIRI: myocardial ischemia reperfusion injury; MyoD-/-: myogenic differentiation factor knockout; N/A: not Applicable; rhVEGF: recombinant human vascular endothelial growth factor; RIPC: remote ischemic preconditioning; SkM: skeletal muscle; SMBs: skeletal myoblasts; TAC: transverse aortic constriction; VEGF: vascular endothelial growth factor; VSMCs: vascular smooth muscle cells;

**Table 3:** The Effects of Skeletal muscle-derived MiRNAs on the Heart and Blood Vessels

Author, year	Sources of MiRNAs	MiRNAs	Models/ Diseases (Methods)	Targeted organs/ cells	Effects on heart/blood vessels
Hiroya, 2022 17	serum of Akt1 transgenic mice	EV-miRNA-206	mice skeletal muscle growth model (Akt1 transgenic mice)	blood vessels	MiR-206 mimics transfected in HUVECs: angiogenesis↑; endothelial cell function↑
	C2C12 mouse myoblasts		Akt overexpression (IGF-1 stimulation)	HUVECs	
Taiki, 2020 23	atrophic limbs and heart of sarcopenic mice	EV-miRNA-16-5p	MIRI mouse model	heart	miR-16-5p plasmid intravenously injected in I/R-TS (-) mice: cardiac repair↓
	miR-16-5p mimics		N/A	NRVMs	miR‑16‑5‑p mimic added in NRVMs: apoptosis↑; autophagy↓
Nie, 2019 27	C2C12 myotube	EV- miRNA-206	hypoxia	HUVECs	Skeletal muscle-derived exosomes stimulated HUVECs: endothelial cell function↑; angiogenesis↑
Chen, 2020 25	temporal muscle	miRNA126-5p	2VO+EMS rat model	vessels	Transfection of temporal muscle with miRNA126-5p agomir played a role in the blood vessels of brain: revascularization↑; perfusion ratio↑
	microRNA-126-5p mimics		N/A	HUVECs	MicroRNA-126-5p mimics added in HUVECs: EC function↑; angiogenesis↑
Wang, 2019 26	muscle satellite cells	Exo-miRNA-26a	CKD model mice (5/6 nephrectomize)	heart	Intramuscular injection of exo-miRNA-26a, containing muscle surface peptides, played the following roles in the heart: cardiac function↑; cardiac fibrosis↓.
João Lucas, 2017 29	skeletal muscle	miRNA-126	obesity rat model	capillary	Skeletal muscle-derived miRNA-126 increasing: capillarity angiogenesis↑

Akt: protein kinase B; CKD: chronic kidney disease; EC: endothelial cells; EV: extracellular vesicle; HUVECs: human umbilical vein endothelial cells; IGF-1: insulin-like growth factor-1; I/R: ischemia and reperfusion; MIRI: myocardial ischemia reperfusion injury; miRNA: microRNA; NRVMs: neonatal rat ventricular myocytes; TS: tail suspension; 2VO+EMS: two-vessel occlusion plus encephalo-myo-synangiosis.

**Table 4:** The Names of Bioactive Molecules from the Skeletal Muscle on the Heart

Effects of bioactive molecules on heart	Names of bioactive molecules
improving cardiac function	EV- VEGF 22; EV-miRNA-26a 26; myoblasts or non-myogenic cells 24; triple-layer SkM sheets 30; VEGF×2 35, 42; skeletal myoblasts and bone marrow cells 37; MyoD^-/-^ and wild-type myoblasts 45; IL-10 34; skeletal muscle and mesenchymal stem cells 41; musclin 19; myonectin 28
decreasing cardiac fibrosis	EV-VEGF^22^; EV-miR-26a 26; musclin 19; VEGF 35; myoblasts or non-myogenic cells 24
reducing cardiac injury or myocardial infarct size	Myonectin 28; IL-10 34; VEGF×2 35, 42; triple-layer SkM sheets 30; MyoD^-/-^ and wild-type myoblasts 45
improving cardiomyocyte contractility	Musclin 19; IL-10 34;
improving aerobic performance	FNDC5 33;
promoting myocardial regeneration	VEGF 35;
Suppressing apoptosis	Myonectin 28; MyoD^-/-^ and wild-type myoblasts 45; VEGF 35
inhibiting inflammatory responses	Myonectin 28; triple-layer SkM sheets 30; skeletal myoblasts and bone marrow cells 37;
reducing histologic rejection in the model of heart allotransplantation	IL-10 38;
disturbing cardiac repair, promoting apoptosis and decreasing autophagy	EV-miR-16-5p 23;

b-FGF: basic-fibroblast growth factor; BMCs: bone marrow cells; EVs: extracellular vesicles; FNDC5: fibronectin type-III domain-containing protein 5; FSTL1: follistatin-like 1; SkM: Skeletal Muscle; IL-10: interleukin-10; miRNA: microRNA; Skm: skeletal muscle; SMBs: skeletal myoblasts; VEGF: Vascular Endothelial Growth Factor.

**Table 5:** The Names of Bioactive Molecules from the Skeletal Muscle on Blood Vessels

Effects of bioactive molecules on blood vessels	Names of bioactive molecules
promoting angiogenesis or capillary growth	EVs×2 27, 44; EV-miR-206 17; EV-VEGF 22; EV-VEGF 32; FSTL1×3 18, 21, 36; miRNA-126 29; VEGF×2 35, 42; MyoD^-/-^ and wild-type myoblasts 45; b-FGF×2, a-FGF 39, 40; skeletal muscle and mesenchymal stem cells 41; myoblasts or non-myogenic cells 24; miRNA-126-5p 25
improving endothelial cell function	EVs×2 27, 44; EV-miR-206 17; FSTL1×3 18, 21, 36; miRNA-126-5p 25; skeletal muscle and mesenchymal stem cells 41; EV-VEGF 22
alleviating neointimal hyperplasia	FSTL1 31;
alleviating atherosclerosis	EVs 43;
protecting against vascular stiffness and senescence and inflammation	EV-FNDC5/irisin 20;
reducing the plaque size and number in aorta	EVs 43;

b-FGF: basic-fibroblast growth factor; EVs: extracellular vesicles; FNDC5: fibronectin type-III domain-containing protein 5; FSTL1: follistatin-like 1; miRNA: microRNA; VEGF: vascular endothelial growth factor.
